# Development of a Novel Spherical Light-Based Positioning Sensor in Solar Tracking

**DOI:** 10.3390/s23083838

**Published:** 2023-04-09

**Authors:** Oğuz Gora, Taner Akkan

**Affiliations:** 1 Vocational School, Yaşar University, Bornova, İzmir 35100, Türkiye; 2 Izmir Vocational School, Dokuz Eylül University, Buca, İzmir 35360, Türkiye

**Keywords:** solar tracking, light sensors, sensor design, spherical sensor, light-based positioning

## Abstract

Tracking of the sun, which increases the efficiency of solar energy production systems, has shown considerable development in recent years. This development has been achieved by custom-positioned light sensors, image cameras, sensorless chronological systems and intelligent controller supported systems or by synergetic use of these systems. This study contributes to this research area with a novel spherical-based sensor which measures spherical light source emittance and localizes the light source. This sensor was built by using miniature light sensors placed on a spherical shaped three-dimensional printed body with data acquisition electronic circuitry. Besides the developed sensor data acquisition embedded software, preprocessing and filtering processes were conducted on these measured data. In the study, the outputs of Moving Average, Savitzky-Golay, and Median filters were used for the localization of the light source. The center of gravity for each filter used was determined as a point, and the location of the light source was determined. The spherical sensor system obtained by this study is applicable for various solar tracking methods. The approach of the study also shows that this measurement system is applicable for obtaining the position of local light sources such as the ones placed on mobile or cooperative robots.

## 1. Introduction

Solar energy is one of the most promising energy sources in today’s world, where clean and sustainable energy production is highly important. Considering the energy outlook of the world and the studies conducted, solar energy will be accepted as one of the primary energy sources among energy production sources in a few decades [1,2,3]. Demand for solar energy is increasing, particularly in nations that want to decarbonize their economies and cut greenhouse gas emissions [4,5,6]. Thus, it is becoming more prevalent in a variety of fields, including architecture, agriculture, smart city applications, and wastewater treatment [7,8,9].

An increase in the demand for solar energy has accelerated the studies on increasing efficiency in solar-powered systems. Despite the efficiency-enhancing studies conducted in solar cell technologies, the efficiency levels of the systems installed with solar panels still have not reached the desired level. Therefore, methods that will increase the total efficiency of solar-powered systems are of great benefit. The tracking of the sun’s position in the sky is one of the technologies used within this context.

Studies on photovoltaic tracking systems and developed technologies were presented in the studies conducted in a wide range by [10,11,12,13]. Within this framework, photovoltaic tracking systems can be classified as active/passive, closed loop/open loop, chronological tracker, intelligent controlled technologies, and by the degree of freedom of axis.

Most of the studies using sensors, Light Dependent Resistors (LDRs), cameras, and specialized sun sensors are preferred. In the studies using LDRs, the positions where the light intensity of the sun in the sky is high are detected by specially positioning these LDR sensors within the installed systems. The positions of LDRs can be placed either in West-East or North-South directions, or in special positions [14,15,16,17,18,19]. Due to utilization of these sensors, tracking of the sun can only be made within certain limits and without producing angular outputs [20,21,22,23,24,25,26].

Use of camera sensors is another sensor approach for the solar tracking systems. Generally, a powerful embedded microcomputer is needed to employ image processing algorithms and camera with fisheye or wide-angle lenses are utilized in order to capture required images [27,28,29,30]. In addition, there are some sun sensors applicable for solar tracking, but they are usually expensive solutions when compared to the previous sensor usages [31,32,33].

In this study, a novel spherical light-based positioning sensor is proposed to locate a light source in spherical angular form. This sensor offers applicability for various control techniques like those mentioned above. The developed sensor can be positioned beside a solar cell to obtain more efficient energy from the sun or beside robots in industrial production to detect the location referenced to a light source. The sun is considered as the light source; but, from a wider perspective, this light source can be any light source emitted in different wavelengths. Light sensors placed on the spherical body in conformity with the spherical angles provided the required sensor data for tracking of the light source. As the advantage of spherical placement of these light sensors, geometric transformations are not needed while interpreting the data.

**Figure 1:**
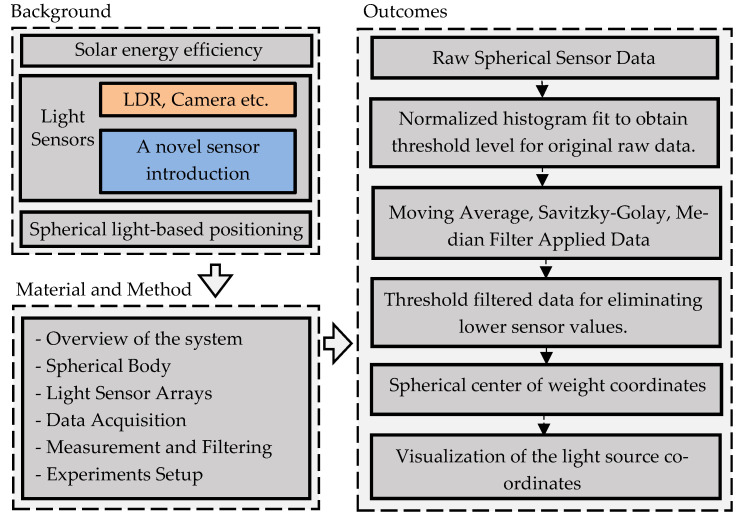
The layout of the study.

The layout of the study is given in Figure 1. In the background section, solar energy efficiency and the sensors used in this field were briefly mentioned. Then, a novel light source localization sensor approach was introduced, which was used for solar tracking or light source localization applications. In the next section, Material and Method was given about the novel sensor idea, sensor construction, data acquisition, filtering, and experimental setup. In the Results section, the outcomes of the study were made. Raw sensor data were processed with a histogram to obtain the threshold filter value, then a smoothing filter was applied. Then, the spherical center was calculated using the filtered data, and the results were displayed as graphical plots.

## 2. Material and Method

### 2.1. Overview of Spherical Measurement System

The designed measuring system consists of a spherical measuring body with light sensors placed on it, circuits designed for measurement, and embedded software architecture. In this measurement system, the beams radiated from a light source fall onto the spherical body. Light sensors were placed on the spherical body in conformity with the spherical angles. An experimental setup was carried out to present a light source and sensor relation. Thus, raw sensor data was collected by the sensor electronics, the multiplexer logic, microcontroller, and the embedded software on the microcontroller. Collected raw sensor data were transferred into the computer environment by means of USB serial communication. With this transferred data, pre-processing of the raw data using filters and light sensor localization calculations can be performed in a personal computer (PC) environment using MATLAB. Moving Average, Savitzky-Golay, and Median filtering were used comparatively to process the raw data in order to better find the coordinates of the light source and obtain more accurate results. After filtering a threshold was applied to ignore the light sensors which has low light data. Furthermore, the center of gravity calculation was used to find the exact light coordinates independent of the exact locations where the sensors were placed. Then raw sensor data, three types of filtered data, and center of gravity coordinates were shown graphically in three-dimensional spherical and two-dimensional top view.

**Figure 2:**
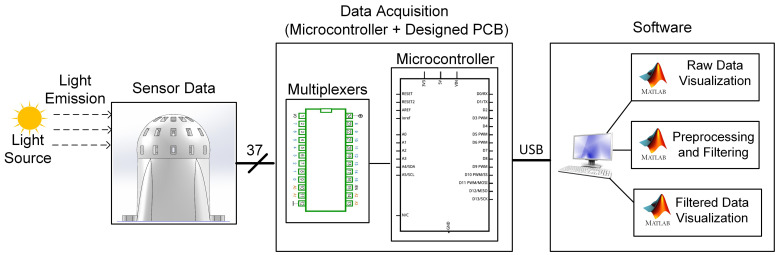
Overview of The Measurement System.

The overview of the sensor measurement system is given in Figure 2 with the hardware and software used for the sensor data processing.

The spherical sensor body collects the reaching light emissions from the light source with the installed 37 light sensors combined with multiplexers and a microcontroller. Simply, the microcontroller reads the light sensors using a multiplexer mechanism and forms a data format which is to be sent. Then, the collected raw light intensity data is transferred by USB serial communication to the PC. In the PC, raw data is preprocessed and filtered using MATLAB software to find the exact light sensors effected by the light source. Then, the raw and filtered data is visualized to test and see the sensor working properly.

The response time of the developed measurement system can be defined as the result of data acquisition, preprocessing, filtering, localization, and visualization. During the data collection phase, the response time of analog circuits and microcontroller circuits was measured as approximately 15 msec. The preprocessing, filtering, and localization processes were measured as a maximum of 25 msec in the MATLAB environment. Thus, the total sensor evaluation time corresponds to 40 msec. Here the fastest filtering algorithm is Moving Average method, with an 8 msec response time. The response time of all processes including visualization is a maximum of 820 msec, using a standard office PC. The measured time varies depending on the processing capacity of the computer used.

#### 2.1.1. Spherical Body

In recent years, especially for prototype development, the high impact of additive manufacturing (AM) or three-dimensional (3D) printing technologies in general has become apparent. Thanks to AM technology, localized, customizable productions of the objects rather than mass production and transportation are made possible. Thus, such parameters as cost and time can be saved on to a considerable extent. AM technology also provides novelties for industrial components that have been traditionally produced. These components, about which common knowledge regarding the mechanical design are already known, can be reproduced with different materials in a customized way [34]. Besides, current designs can be updated with brand new features with the addition of circuit components [35]. Additionally, AM technology contributes to sensor production as well. It is seen in the conducted studies that this technology made contribution in such fields as electrical, thermal conductivity, radiation protection, flow, force, and pressure measurement [36,37,38,39]. This contribution is made by enabling production in different geometries, being easily customizable and used in microscales.

**Figure 3:**
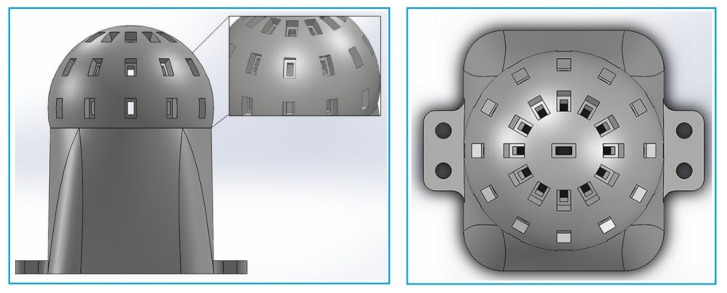
Sensor model front view (**left**), Sensor model top view (**right**).

In this study, a CAD model of the spherical body was designed in a computer and then manufactured by using AM technology as a prototype. For the light sensors, gaps are created on the spherical body in horizontal and vertical directions and with 30° angular distances to enable the light sensors to fit in. This 30-degree aperture in half sphere corresponds 36 light sensors, and we obtain 37 light sensors involving the light sensor located on the top of the dome. The produced model can be seen in Figure 3, as a three-dimensional front view and top view, to understand the spherical body installment of the sensor body. This structure can be flexibly updated and is designed to be used for more light sensors.

The designed model was sliced with the Cura software and printed using PLA filament material with a 0.4 mm nozzle diameter using the Ender 5 3D printer.

**Figure 4:**
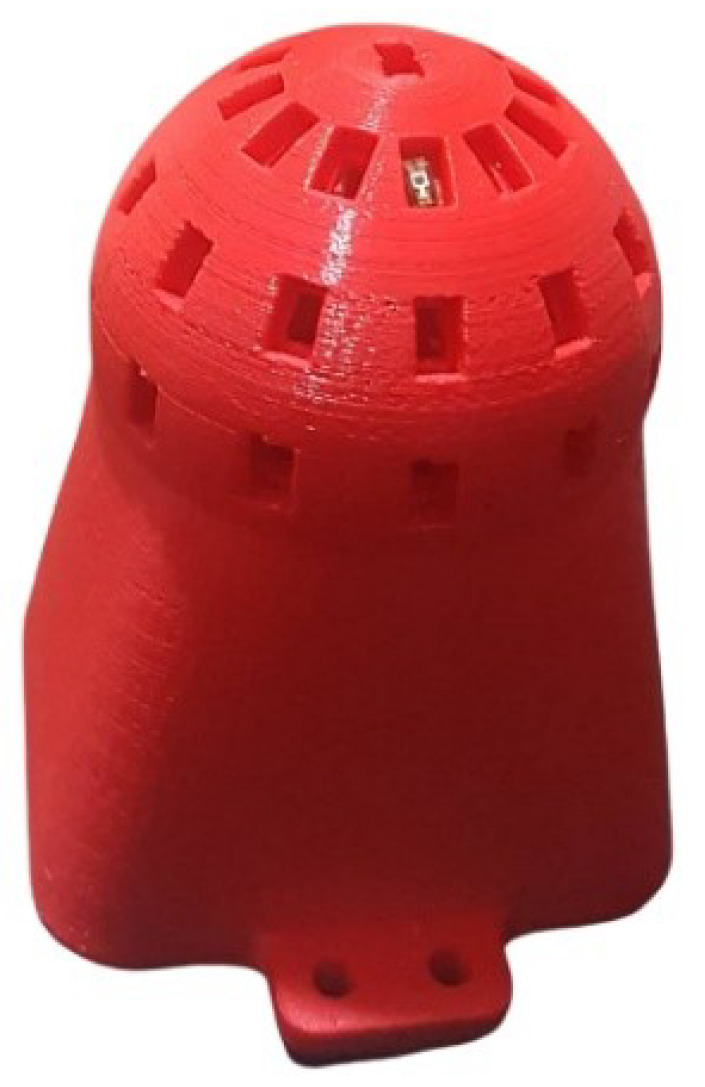
3D Printed body.

By considering the printing quality, printing was completed with the help of supportive structures. In the post-processing phase, supportive structures required for the printing were taken apart and the printing was cleaned up. Then the rasping process was applied manually on a limited extent to obtain a smoother surface. The diameter of the model sphere is 50 mm, the inner thickness for the sphere is 5 mm, and for the part into which the sphere is placed it is 10.92 mm. The inner part of the sphere is left empty. In this way, enough space to place the light sensor cables onto the circuit cards is acquired. The body is affixed to the circuit box underneath with screw holes on both sides to make the body stable and balanced during the measurements. The 3D printed body created based on the designed model and used during the rest of the study is shown in Figure 4.

#### 2.1.2. Light Sensor Arrays and Their Placement

LDR’s can be used for detecting the sun’s position in the sky during solar tracking. In these studies, LDR’s are placed in specific positions in a way to represent the geographical directions with various methods. Although solar tracking is made possible by using LDR’s in this way, we had mentioned that the results are obtained within certain limits and the angular outputs could not be attained.

In our study, instead of LDRs, TEMT 6000, a phototransistor sensitively working especially on the visible wavelengths, is used as the light sensor. Thus, electrical current is obtained with a linear curve in accordance with Lux, the luminous intensity unit emerging in the light sensor. These light sensors exhibit the sensitivity peak point value at 560 nm and the range of spectral bandwidth is 360–970 nm [40]. Concordantly, sun’s solar spectrum emits a major part of its energy in the visible light range of 350–750 nm [41]. These values show that the measurements made by means of these light sensors are appropriate to use for detecting the light emitted by the sun in the sky.

**Figure 5:**
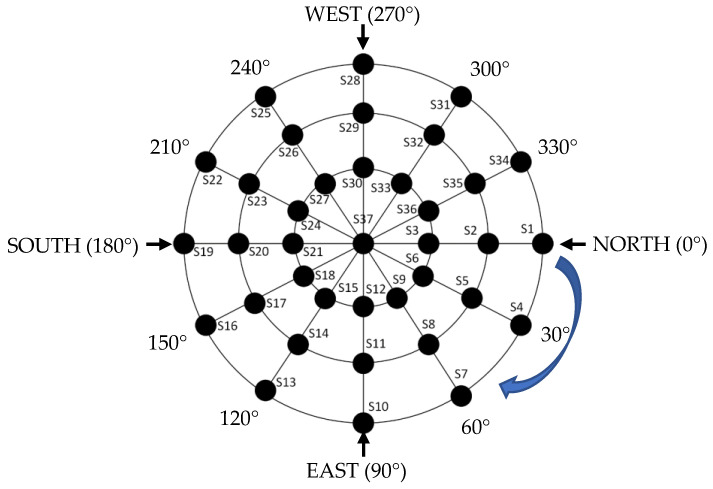
Top view of the locations of 37 light sensors.

In the study, light sensors are placed on the spherical body with the distance of r = 25 mm fixed radius value and used in array order. Top view of the locations of 37 light sensors is shown in Figure 5.

Examining the installment of the light sensors in the spherical structure, 30°-degree angular spacing is created among the light sensors in azimuth direction and in elevation direction. The light sensors are numerated with the symbols of S1, S2, S3, S4, … S37. The enumeration starts from the 0° azimuth and 0° elevation (S1) and continues in the elevation direction with 30° elevation (S2), 60° elevation (S3), then S4 comes in 30° azimuth and 0° elevation and continues like that. The S37 is in the top of the sphere in 90° elevation in every azimuth angle.

#### 2.1.3. Data Acquisition

Data acquired from the light sensors, placed on the spherical body, compose the raw sensor data. TEMT 6000 light sensors used in the spherical sensor produce linear current output in the range of 10–1000 lux. These values, which are read as voltage values by the ATmega328P microcontroller, are read in the range of 0–5 V with 10-bits analog to digital conversion resolution. Voltage output values (0–5 V) of 37 light sensors are read by the designed multiplexer circuits.

Reading procedure is carried out by sequentially reading the light sensors connected to three multiplexers. The voltage values read by the light sensors are transformed into luminous intensity (lux) values by the microcontroller. Then data transferred serially using a serial-USB adapter to PC MATLAB environment via USB interface. In order to prevent possible speed and synchronization problems, the data to be transferred is optimized in terms of data verification.

In the empirical experiments carried out, optimal serial communication speed is determined as 500 kbps (~62.5 kb/s) considering the transfer capacity of the microcontroller. Freezing and lagging problems are observed under this value during the MATLAB visualization process. On the other hand, when the study is carried out above this level, loss can be seen in the transferred data. Transferred data are checked by the Checksum method from both communication nodes and the data are proved to be error-free. In addition, in order to protect the stability of the transferred data load, balanced data flow is provided by sending the same amount of data bit during each transfer process.

#### 2.1.4. Data Filtering

The raw sensor data is plotted graphically for different azimuth and elevation angles to understand how much the reference light sensor position differs from the position measured in Experiment 1. To reduce the difference, the raw sensor data should be filtered for better sensor results. Experiment 1 setup and related graphs are given in the following sections.

Experiment 2 was designed for filtering purposes and three different smoothing filters were tested to filter the raw sensor data and the improved results are presented in the following sections. For this purpose, Moving Average (MA) filter, Savitzky-Golay (SG) filter, and Median Filter (MF) were used for noise reduction or smoothing out the short-term fluctuations in a data set. Thus, high-frequency or ambient noise components were filtered from the data coming from the sensors. However, these filters work in slightly different ways and have different strengths and limitations.

The MA filter is a simple filter that uses the measured data sequentially for averaging [42,43]. The filter calculates the average of the adjacent data elements and replaces these elements with the average value calculated. This operation results in a smoothing action and reduces the noise. The disadvantage is that it lags behind the real time data because of the averaging calculation. The following Equation (1) shows the calculation of the MA filter with using measured data. In the equation *X* denotes measured data samples and n denotes amount of measured data for averaging.

(1) $$X_{\mathrm{MAF}}=\frac{X_{1}+X_{2}+\ldots X_{n}}{n}$$

The MA filter used in the study; 40 measurement samples were used in order to take average for each filtering window. The 40-point MA filter algorithm used in this study is given in Algorithm 1.
**Algorithm 1:** 40 Points—Moving Average Filter Algorithm1:  Define A[] = 0, sum = 0, i = 1 2:  for each sensor_value do 3:    do until i = 40 4:      Measure S_i_
5:      sum = sum + S_i_, i = i + 1 6:      For i = 40, A[] = sum/40 7:      end 8:    repeat for next 40 sensor_value 9:  end for sensor_value

The Savitzky-Golay Filter is introduced as a smoothing algorithm [44,45]. The filter fits a polynomial to a set of adjacent data elements and replaces each element with the value of the polynomial at that point. When it is designed well, it can be more successful than the moving average filter. It smooths the signal while preserving features such as peaks. It is particularly used for smoothing signals that have noise components or unwanted variations. Low-latency and low-frequency response loss are the advantages, but it has lower noise reduction performance. In the algorithm a set of integers (a) are derived to use as weight coefficients. These coefficients fit the data to a polynomial, the filter out (y) as a series of $y_{i}$s can be calculated using the following Equation (2).

(2) $$y_{i}=\frac{\sum_{k=-n}^{n}a_{k}y_{i+k}}{\sum_{k=-n}^{n}a_{k}}$$

Savitzky-Golay filtering calculations are given in Algorithm 2. Additionally, more enhanced and superior versions are available [46,47].
**Algorithm 2:** Savitzky-Golay Filter Algorithm1:  Define data, data_length, window_length 2:  for i in range data_length 3:    window = data[integer(i—data_length/2): integer(i + data_length/2 + 1)] 4:    coeffs = polynomial fit in range of window_length 5:    poly = define polynom using coeffs 6:    y[i] = poly(integer (data_length/2)) 7:  end

Median filter is another filtering and de-noising algorithm used in signal processing applications [48,49]. The filter finds the median of a data set for the smoothing action. Median filter preserves the data important peak value features, resists the effects of outlier values in the data, and is good at removing the rare events. This preserving action and rare event removing are suitable for our sensor application. However, they can be computationally slower than the other smoothing filters. The algorithm for the 5th order MF is given in Algorithm 3.
**Algorithm 3:** Order = 5 Median Filter Algorithm1:  Define Y[] = 0, i = 1 2:  Measure light sensors array S[] 3:  for each 5 light_sensor_values (S_i_, S_i+1_, S_i+2_, S_i+3_, S_i+4_) do 4:    Sort light_sensor_values 5:    Select median 6:    Assign Y_i_ to median value, i = i + 1 7:  end for 5 light_sensor_values 8:  repeat for next light_sensor_values

A histogram was applied using 37 light sensors data to find maximum point of the curve which is useful to calculate the threshold value for discarding the light sensors has lower light intensity value. The calculated threshold values are given in the Results section and applied before the filtering methods to get more accurate sensor localization data. The threshold value calculation algorithm is given in Algorithm 4.
**Algorithm 4:** Threshold algorithm1:  Define sensor_data[], mu, sigma 2:  total = mu + sigma 3:   maxvalue = find max (sensor_data[]) 4:   thrvalue = total / maxvalue

The result localization sensor data were processed with the center of gravity angles calculation to find a theoretical light source coordinates as a point. Both angles were simply calculated as a ratio of each light sensor angle weighted sum over total 37 light sensor values. The equations are given to find the elevation and azimuth angles of the center of gravity in Equation (3) and (4) derived from the study [50] considering the distance form of center of gravity equations.

(3) $$\theta_{cog}=\overset{37}{\underset{i=1}{\sum }}\theta_{i}\cdot S_{i}\;/\;\overset{37}{\underset{i=1}{\sum }}S_{i}$$

(4) $$\varphi_{cog}=\overset{37}{\underset{i=1}{\sum }}\varphi_{i}\cdot S_{i}\;/\;\overset{37}{\underset{i=1}{\sum }}S_{i}$$

After finding the center of gravity azimuth and elevation angles, the Cartesian coordinates can be calculated using the conversion Equations (5)–(7); therefore, the spherical center of gravity point is easily marked on the spherical graphics to determine the light of source localization. This conversion can be easily done by using sph2cart() function of MATLAB.

(5) $$x=r.*\cos\left(elevation\right).*\cos\left(azimuth\right)$$

(6) $$y=r.*\cos\left(elevation\right).*\sin\left(azimuth\right)$$

(7) $$z=r.*\sin\left(elevation\right)$$

Spherical sensor raw data filtered—threshold applied processed data and center of gravity can be visualized and the related plots given in Section 3.

### 2.2. Experiments Setup

**Figure 6:**
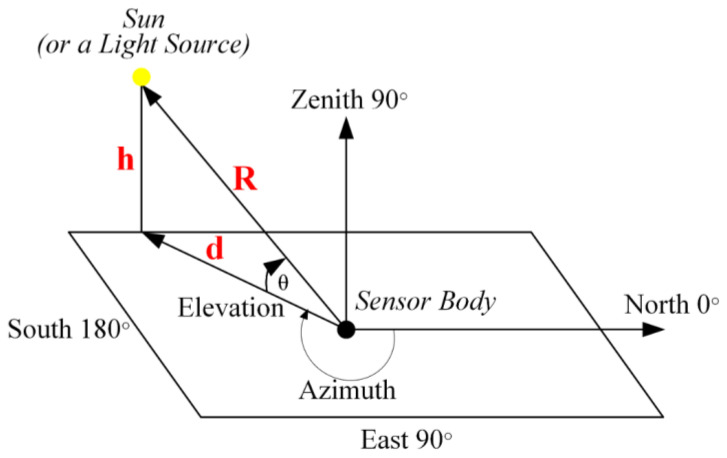
Relationship Between Sun and Reference Point.

Azimuth and elevation angles are the angles used for determining the sun’s location in the sky. Azimuth angle (φ) states the angle that is formed by the sun’s position and the geographical north while the elevation angle (θ) points out the angle that the sun forms with the horizontal plane. The relationship of the sun in the sky with the observer reference point is shown in Figure 6.

The proposed measurement system was tested in the experiment environment and measurement values that are corresponding to the reference values were aimed to be obtained. Artificial light source was used in the experiments conducted and location of the light source was changed and thus, measurements were maintained and recorded. Subjects paid attention to regarding the experimental setup design and some key points were as follows:

- On condition that the measurement system remains at the fixed position, the measurements were made by moving the used light source in azimuth and elevation angles and with 30° angular distance. The measurements were made for 37 different locations in total.
- The distance between the measurement system and the light source (d) was fixed at 40 cm value. The reason is that h and d values increase more than necessary depending on the elevation θ angle value.
- Required h, d, and R parameters were calculated with the trigonometric features in the measurements, and they were determined by using a laser meter in each measurement. The direction of the light source was controlled with a laser. Thus, erroneous measurements were avoided even though the light source was manually placed at the elevation and azimuth angles.
- Spherical body and the electronic box used in the measurement system were placed in a way to enable independent movement in azimuth direction. By designing the experimental setup in this way, angular rotation of the measurement system was ensured without needing the movement of the light source in different azimuth angles.
- Experimental environment was isolated from interfering and external light sources during the experiments.

**Figure 7:**
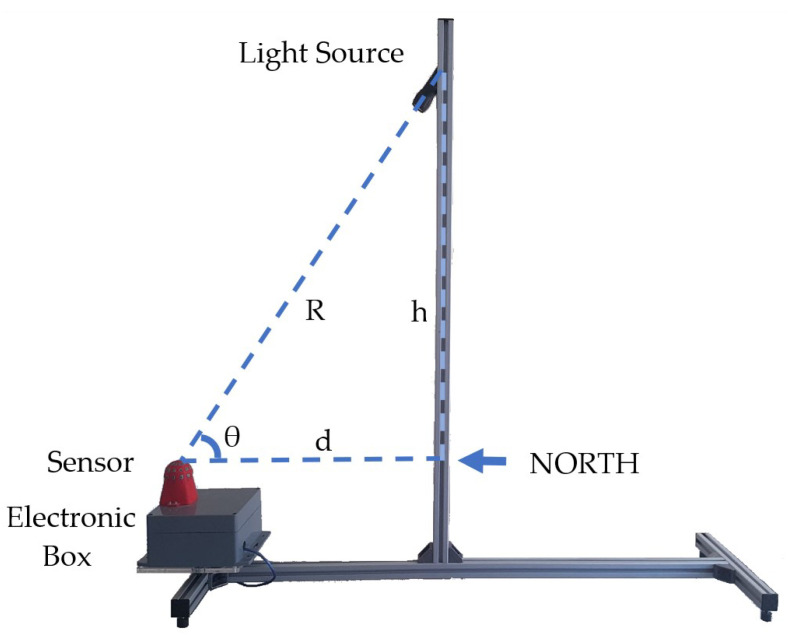
Experimental Setup for the novel sensor test.

According to key points mentioned, the experimental setup shown below in Figure 7 was built and used during the rest of the study. In this experimental setup the mechanical experiment system was built using aluminum profiles connected with profile connectors. The adjustable plastic legs were needed to ensure the system is parallel to the ground. The light source was fixed to the perpendicular profile and directed to the sensor attached to the end of the horizontal profile.

The electronic box contains the necessary electronic system to operate the sensor and get the sensor data to a PC via USB connection. The electronic box can be rotated to adjust the sensor azimuth direction.

Using the experimental setup two different experiments were carried out to prove the sensor operation success.

Experiment 1: A LED light source was directed to selected light sensors to test the intended light sensor values. Here the aim of the experiment is to find which light sensor is affected by a light source and shows the maximum light intensity value. The measured maximum value has a close neighborhood with the reference target light sensor. The parameters are shown in Table 1.

The total six measurements were conducted at these locations and the results of sensor data recorded as raw .txt data which labeled as measurement number (MN) for Experiment 1. The measured sensor location difference and the sensor values in the neighborhood were displayed in Results Section 3.1

Experiment 2: A LED light source was directed to light sensor groups aiming one of them in six designed measurements to apply filtering to refine the light sensor outputs and to calculate the center of gravity. Here the light source distance was higher than the one in Experiment 1. The reference light sensors were selected as a group of neighbors to find out how the sensor system differentiates these neighbors from each other. The parameters related to the measurement numbers for Experiment 2 are given in Table 2.

The light source was positioned in relation to the distance parameters of d, R, and h. The first four experimental measurements were aimed to test S1, S2, S3, and S37 respectively, only changing the elevation angle from 0° to 90° degrees and keeping the azimuth angle fixed at 0°. The last two measurements were aimed to test S35 and S32, respectively, turning the table in azimuth direction from North to East with keeping the elevation angle as 30° and moving azimuth angle from 330° to 300°.

The total six measurements were conducted at the mentioned locations and the results of sensor data were recorded as raw .txt data which labeled as measurement number (MN) for Experiment 2. The histograms plots, three-dimensional, and two-dimensional sensor graphical visualizations of the measurements were given in the Results Section 3.2.

## 3. Results

### 3.1. Raw Data Measurements for Experiment 1

**Figure 8:**
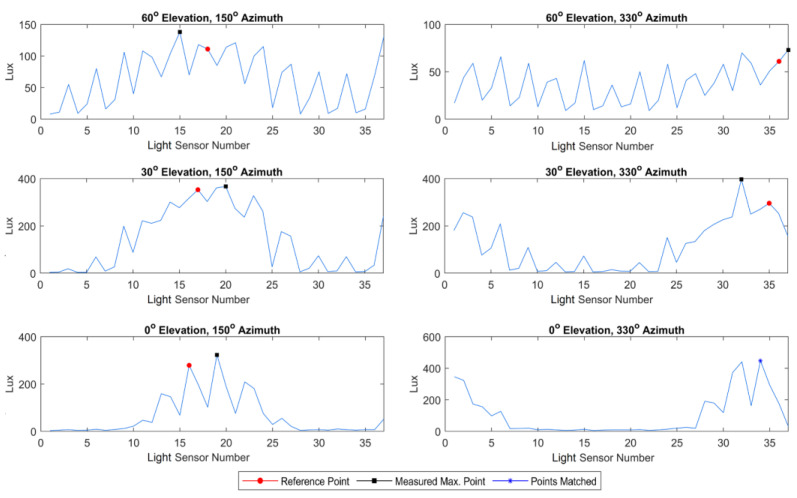
Selected samples of measurement results for Experiment 1.

Raw data measurements for Experiment 1 were carried out using the experimental setup mentioned in Section 2.2. The measurements had a total of 37 light sensors at six locations; data are shown in Figure 8.

Here, the measured data were shown as raw data values without any processing. Elevation and azimuth angles, at which the light source is located, were accepted as reference points. Measurement results yield the highest outputs mostly around reference point or their neighbors. It is seen that the light was scattered, and the light sensors created approximate values with neighbor points.

In the first three measurements, the azimuth angle was kept constant at 150° and the elevation angle was changed from 60° to 0°. In these three measurements, the position of the light source was decreased in terms of the elevation angle. Thus, it was seen that the light beams scatter more, and the total measured values decrease. The reference point and the measured point which has the maximum value was at a close neighborhood but not at the same point. In the last three measurements, the azimuth angle was kept constant at 330° and the elevation angle was changed from 60° to 0°. Here, again the reference point and the measured point location are at different points, but they are closer each other than the first three measurements. In some cases, a match can also occur, such as at 0° altitude and 330° azimuth. This shows that different measurement accuracies are possible because of the light source being manually adjusted. This can be fixed either through an automatically controlled experiment setup or by using filtering and mathematical calculations to find the exact localization points as in Section 3.2.

### 3.2. Preprocessed and Filtered Data Measurements for Experiment 2

**Figure 9:**
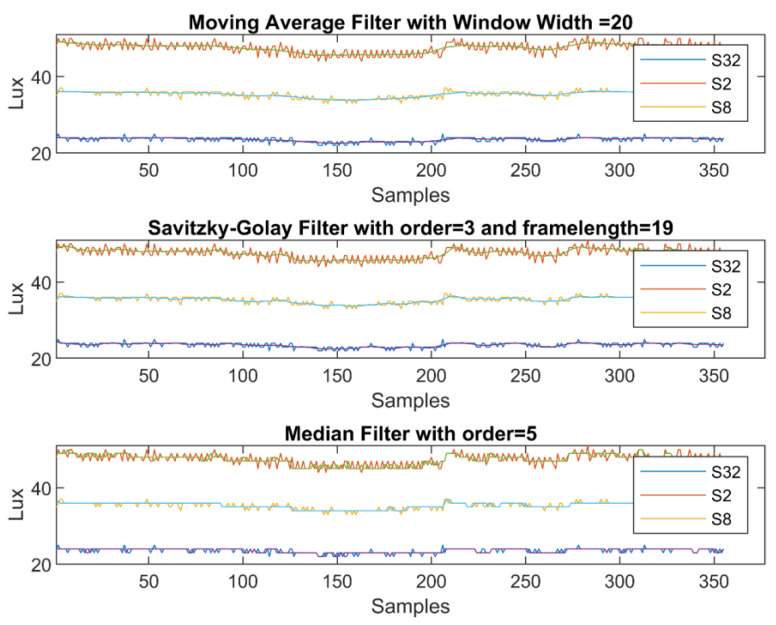
The smoothing action of the filters for selected S2, S8, and S32 sensors.

According to the filtering methods mentioned in Section 2.1.4, collected data were processed and noise components of measurements were eliminated successfully. The neighboring light sensors on a line of S2, S32, and S38 were selected to show all the 4 s of data (355 sample) while the light source was directed to the location of the spherical sensor at zero degree of azimuth angle and zero degree of the elevation angle. The smoothing action can be seen for three filtering algorithms results for selected S2, S32, and S38 sensors in Figure 9.

**Figure 10:**
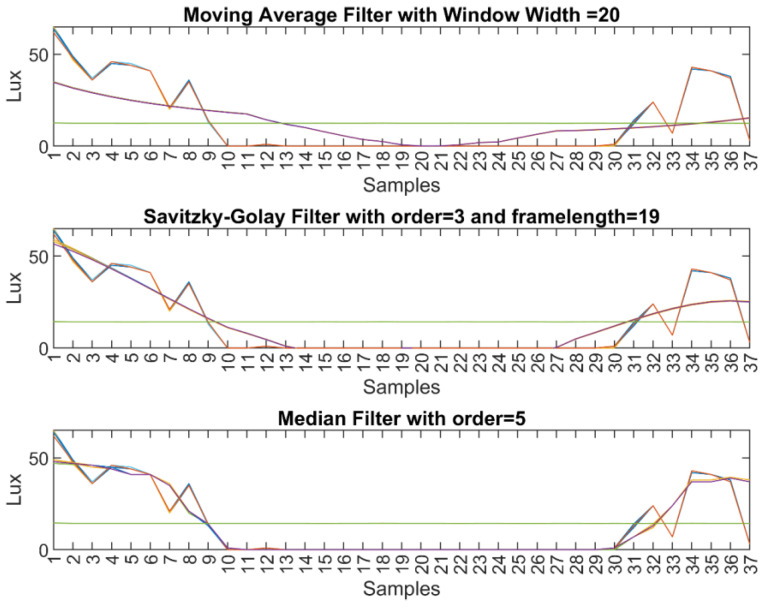
All 37 light sensors data filtering results for different methods.

For three filtering methods, all 37 light sensors data were plotted to show light sensors value when the light source was spotted directly to the location of S1 at an elevation of 0 degree (Figure 10).

Especially the zero valued sensor data were really considered to be removed for finding the right threshold value. The average value of 12 was nearly the same for all the filters mean value. This is the discarding low limit of the sensor data which is shown in Figure 10 with a green line.

From Figure 10, it can be clearly seen that the Median filter has the fastest response and more successful results amongst other filtering methods proposed.

### 3.3. Visualization of Measurements for Experiment 2

Six different measurements were designed in which the experimental light source was aimed at different sensor regions to see and test how the sensor works. The collected sensor data of these measurements were processed using MATLAB. First, a histogram was applied to all of the 37 light sensors data to see the illumination range of the light sensors and the histogram distribution. Using normal distribution fitting on the histogram data, the threshold parameter was calculated. The threshold value was applied to the raw data for eliminating the sensor data which do not contribute much. The calculated threshold value ensures that the light source localization is more reliable and correct. Then, three selected filtering methods were applied to raw data to obtain a more precise localization result.

The histogram was applied with MATLAB histfit function (histogram fitting) with normalized y data. This function draws a histogram of the values in the data by creating several bins equal to the square root of the number of items in the data and then fits it to a normal density function. After, MATLAB fitdist function (distribution fitting) fits a normal distribution to sensor data, and mean value and standard deviation parameters were calculated. μ shows the mean value and therefore maximum point of the fitted histogram curve. This can be a candidate for a threshold value considering the maximum sensor value. However, here a higher value which corresponds to the 70.7% of the maximum value was preferred. σ shows the standard deviation and the final threshold value is here calculated as sum of histogram μ and σ values. Using the ratio of final threshold value over maximum sensor value, we get the normalized threshold value to apply a threshold filter. The above processing steps were repeated for every measurement and related threshold values were given for each of the measurement numbers (MN1 to MN6) in Table 3.

**Figure 11:**
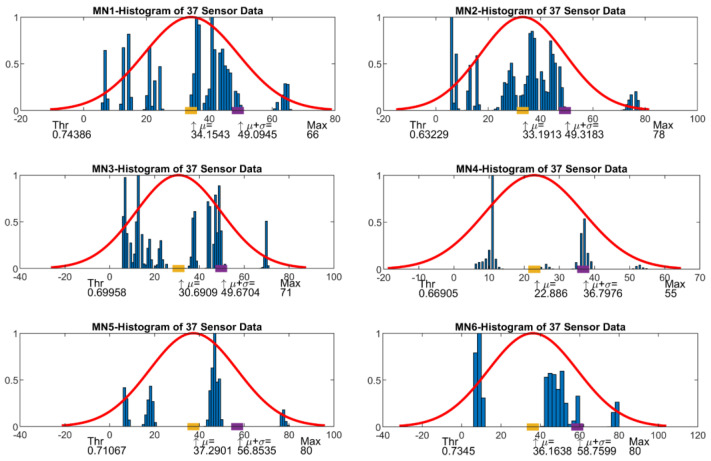
Histograms for measurements MN1 to MN6.

The histogram plots were given in Figure 11 for MN1 to MN6. The histogram plot was calculated for all 37 light sensors data to define threshold value for each measurement. In the histogram plots also, the needed parameters mean value (μ), the sum of mean and sigma value (μ + σ), maximum value, and calculated threshold values were printed under the graphic.

Two display formats were preferred to visualize the data obtained with the measurements. The first one was the representation based on spherical coordinates; the second one was the top view to see 360-degree light sensors positions from top. For a better understanding and to see the sensor experiment results, the NORTH was shifted 180-degree in clockwise. Therefore, S1-S2-S3 was now in the place of S19-S20-S21, and S19-S20-S21 was in the place of S1-S2-S3. Yet still the light sensors placement direction was the same as clockwise. In these plots also the minimum and maximum values of light intensity values with color codes were given in color bars. Referring to these lux color codes the light sensor values were shown in 3D sensor locations. The top view was also given to perceive all sensor values in the spherical form. Finally, the calculated center of gravity was marked with a black dot in each of the 3D and top views.

**Figure 12:**
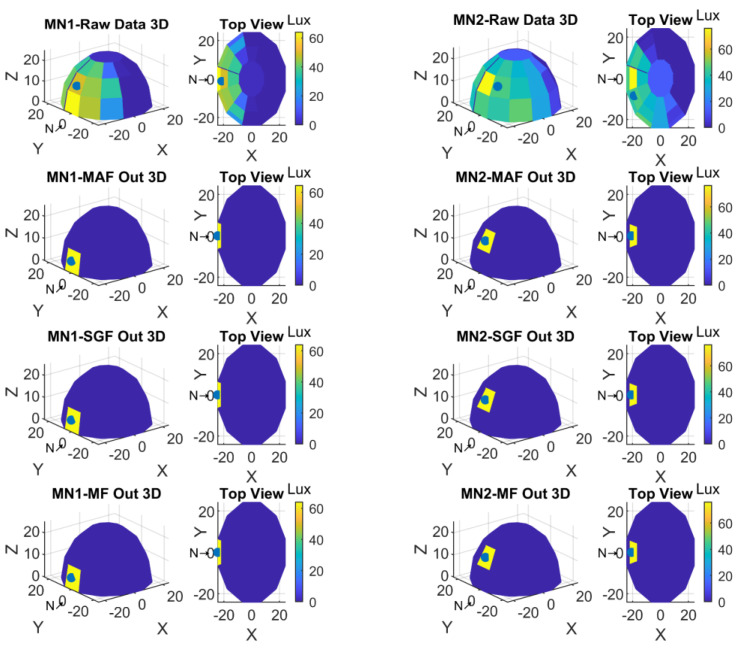
Spherical and top view of MN1 and MN2 experiment result.

The Raw Data, Moving Average Filter output, Savitzky-Golay Filter output, and Median Filter output plots in three-dimensional hemisphere format and in top view format were given in Figure 12 for MN1 and MN2 measurements.

**Figure 13:**
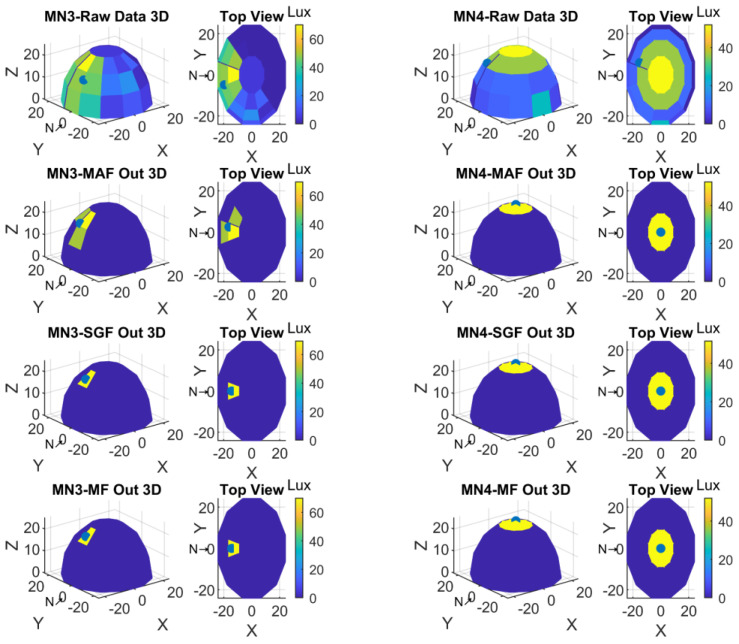
Spherical and top view of MN3 and MN4 experiment result.

Graphic plots for the measurements from MN3 and MN4 were shown in Figure 13.

**Figure 14:**
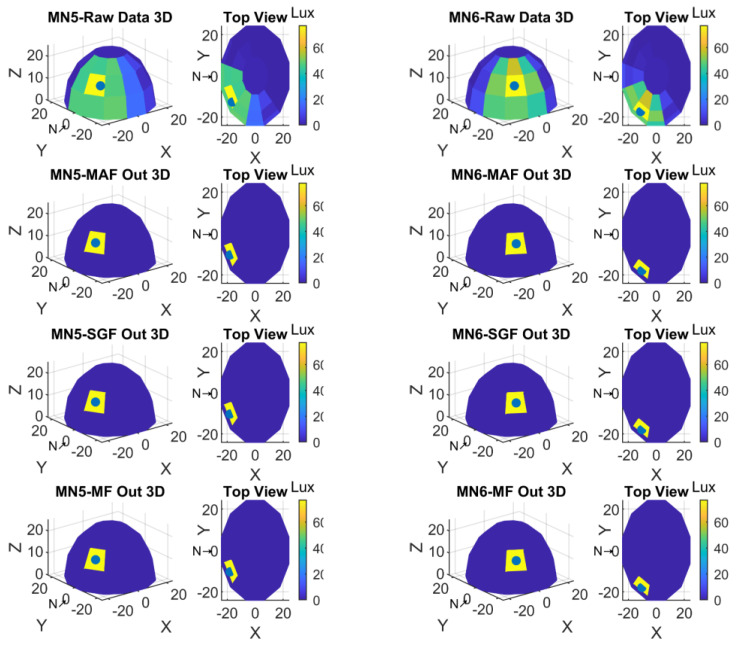
Spherical and top view of MN5 and MN6 experiment result.

Graphic plots for the measurements from MN5 and MN6 were shown in Figure 14.

Although three methods described for smoothing of the sensor data, further research can be done on different techniques to get better results. Here all filters perform nearly the same. However, median filter produces more reliable results.

## 4. Conclusions

There have been many studies on solar tracking systems and sun sensors in the literature. These studies are focused on various problems of solar energy systems. Collecting the highest energy from the sun is one of these problems. To achieve that, the position of the sun in the sky must be determined accurately.

Many methods have been proposed that provide a solution to this issue. LDRs have been frequently used as a sensor circuit element, especially in the early studies. Although LDRs are preferred because they are cheap and easily available, it is not possible to build high accuracy systems with them. Besides, systems that use geographic location and time-date information also achieve good results. In these systems, there is a need to make astronomical calculations or to provide these data from external sources. Obtaining these data by calculating instantaneously brings with it high computational needs in the area where these systems are located. With the development of sensor technologies, technologies such as MEMS (Micro-electro-mechanical Systems), in which microelectronic circuits are used, have also contributed to the field of solar energy. These technologies have provided high accuracy data to be obtained, but the difficulty of production and high cost prevent their widespread use. In addition, image processing technologies have also become one of the solutions that require intensive computation in order to establish high-accuracy systems in this field.

When all the proposed methods are examined, it is seen that each of them has advantages and disadvantages. In this context, it is understood that there is a need for cost-effective and highly accurate solutions. In addition, it stands out as a desired feature that these solutions do not require intensive calculations and detailed geographical information. In this study, we intended to contribute to filling this gap in the literature. The aim was to find a solution for finding the position of the sun in the sky. The work in this study focuses on designing a spherical solar positioning sensor with high accuracy that can be used in solar tracking systems. In the first part of the study, a 3D sphere model was created in a computer aided design software to take advantage of spherical data collection. In order to obtain the data, appropriate light sensors were determined, and studies were carried out to realize the optimum use in this design. In this context, it was determined that the most appropriate sensor angular interval should be 30° for a sphere with the determined diameter. Accordingly, the light sensors were placed in the spherical prototype model. Then, electronic circuits that transmit the data measured by the sensor to the computer environment were designed.

The novel sensor proposed in this work is an easily fabricated spherical structure with light sensors spaced at appropriate intervals. Thus, unlike any planar light sensor (LDR, photodiodes, phototransistors, etc.) or camera sensor structure (CCD, lateral photodetector, etc.), it does not require the addition of a lens for angular position sensing and finding the position of the incident light in spherical coordinates. In addition, since the position of the light source is detected spherically, there is no need to apply any geometric transformation. Thus, a light source positioning sensor that can be designed in hemispherical or fully spherical form for the intended use, where the number of sensors and the sensor light detection wavelength can be selected, has been obtained. This sensor is simple to manufacture, low cost, easy to use and can be used not only for locating light sources such as the sun but also for locating light sources in industrial production sites.

The transmitted raw data had noise components. In order to avoid these noisy components of raw data and extract significant data, Moving Average (MA), Savitzky-Golay (SG), and Median (M) filters were applied. As an algorithm complexity MA and M filters are easier to implement then the SG filter. For low-pass filtering all filters perform similarly, but Median Filter was consistent in all measurement examples and easy to implement. Therefore, Median Filter was selected as a filter. The filtering method and the parameters can be properly selected with further new experiments in the future works.

Then, visual image representations related to the tracking of the light source were obtained. These visual images can be processed to find the light source localization, but the processing power is needed. As a solution, the coordinates of the light source were calculated to get a more accurate and numerical result using center of gravity calculations. The center of gravity algorithm was implemented in azimuth and elevation coordinates for 37 independent light sensors to find spherical coordinates. Every light sensor independently has its center of gravity is on its center points, but the resulting center of gravity output can be any point on the sphere’s surface. Using the center of gravity result spherical coordinates; the microcontroller, programmable logic controller (PLC), or a PC can gather the localization coordinates numerically from the novel sensor designed. Since the sensor works by using the center of gravity algorithm to localize the sun according to the highest light intensity values falling on the light sensors, it also works accurately in cloudy or shady weather conditions. Cloudy or shady weather conditions are known to adversely affect the accuracy of the system in solutions where diffuse radiation is not taken into account or where the sun’s position is found only by the chronological method.

The final system has a resolution depending on the number of sensors used. The selected light sensor numbers as 37 (which was designed to correspond 30-degree aperture in both azimuth and elevation angles) is at a level that can accurately track the source in solar energy applications basically. In addition, the center of gravity calculation allows us to have more resolution as a spherical localization to obtain rather than the discrete exact sensor localizations. However, considering the different usage areas of the sensor—for example in robotics—it may be necessary to adapt the large number of sensors. This will cause an increase in the complexity of the system as well as the software load and the data transfer size will be scaled up. However, because of the scalability of the sensor construction this can be easily implemented by changing the mechanical design.

The sensor acts as a specifically designed spherical camera sensor which does not need any spherical lens attached to a planarly oriented camera, as we are accustomed to. Therefore, the spherical mathematical conversion is not needed to localize the light source position in a three-dimensional space. Thus, the natural three-dimensional structure of the studied novel localization sensor will have a software load advantage.

Future academic studies or utilization in real life light localization problems can be inspired by this study. In addition, the potential of the study will lead various research in sensor design, solar energy, and robotics applications. Some of the possible future works are listed below.

- The sensor test was done with a manually adjusted mechanical testbed. The sensor orientation was also adjusted with hands. For more accurate tests an automatic motor driven testbed can be built.
- This study can be made more compact and standalone and can be turned into a smart sensor that can be used directly at the point where it is positioned with appropriate processing capacity and can be directly integrated into solar tracking systems. Several smart sensors can be deployed in an internet of things (IoT) environment for the smart interconnected applications.
- The studies that can be done on the production technology of the sensor computer aided model can be transformed into a more advanced sensor where more light sensors can be used together and can be optimized in terms of sensor placements. Thus, the resolution of the sensor can be increased.
- The spherical sensor structure will be able to find new areas of use in indoor environments with light sensors that will operate at different wavelengths. For example, it can be used for location coordination of mobile robots working in dark factories which yields energy saving.
- Fuzzy logic, neural network, or deep learning algorithms can be used to increase the performance of the sensor in the light source localization or light source tracking process.

## 5. Patents

The spherical sensor, which is one output of the study, was resulted with a national patent and applied for the international patent.

## Figures and Tables

**Table 1:** Parameters of Measurements for Experiment 1.

Experiment 1 Measurement Number (MN)	d (cm)	R (cm)	h (cm)	Corresponded Reference Light Sensor	Elevation Angle (θ)	Azimuth Angle (φ)
1	20	20	0	S_18_	60°	150°
2	20	23.09	11.54	S_17_	30°	150°
3	20	40	34.64	S_16_	0°	150°
4	20	-	-	S_36_	60°	330°
5	20	23.09	11.54	S_35_	30°	330°
6	20	23.09	11.54	S_34_	0°	330°

**Table 2:** Parameters of Measurements for Experiment 2.

Experiment 2 Measurement Number (MN)	d (cm)	R (cm)	h (cm)	Corresponded Reference Light Sensor	Elevation Angle (θ)	Azimuth Angle (φ)
1	40	40	0	S_1_	0°	0°
2	40	46.19	23.09	S_2_	30°	0°
3	40	80	69.28	S_3_	60°	0°
4	40	-	-	S_37_	90°	-
5	40	46.19	23.09	S_35_	30°	330°
6	40	46.19	23.09	S_32_	30°	300°

**Table 3:** Distribution Parameters and Threshold Values of Measurements.

Experiment 2 Measurement Number	μ	μ + σ	Maximum Value	Normalized Threshold Value
MN1	34.153	49.0945	66	0.74386
MN2	33.1913	49.3183	78	0.63229
MN3	30.6909	49.6704	71	0.69958
MN4	22.886	39.7976	55	0.66905
MN5	37.2901	56.8535	80	0.71067
MN6	33.1913	49.3183	78	0.63229
